# Genomic chronicle of SARS-CoV-2: a mutational analysis with over 1 million genome sequences

**DOI:** 10.3906/biy-2106-8

**Published:** 2021-08-30

**Authors:** Osman Mutluhan UĞUREL, Oğuz ATA, Dilek TURGUT-BALIK

**Affiliations:** 1 Department of Bioengineering, Faculty of Chemical and Metallurgical Engineering, Yıldız Technical University, İstanbul Turkey; 2 Department of Basic Sciences, School of Engineering and Natural Sciences, Altınbaş University, İstanbul Turkey; 3 Department of Software Engineering, School of Engineering and Natural Sciences, Altınbaş University, İstanbul Turkey

**Keywords:** SARS-CoV-2, COVID-19, mutation, variation, genomic analysis, single nucleotide variant (SNV), 1 million genomes

## Abstract

Use of information technologies to analyse big data on SARS-CoV-2 genome provides an insight for tracking variations and examining the evolution of the virus. Nevertheless, storing, processing, alignment and analyses of these numerous genomes are still a challenge. In this study, over 1 million SARS-CoV-2 genomes have been analysed to show distribution and relationship of variations that could enlighten development and evolution of the virus. In all genomes analysed in this study, a total of over 215M SNVs have been detected and average number of SNV per isolate was found to be 21.83. Single nucleotide variant (SNV) average is observed to reach 31.25 just in March 2021. The average variation number of isolates is increasing and compromising with total case numbers around the world. Remarkably, cytosine deamination, which is one of the most important biochemical processes in the evolutionary development of coronaviruses, accounts for 46% of all SNVs seen in SARS-CoV-2 genomes within 16 months. This study is one of the most comprehensive SARS-CoV-2 genomic analysis study in terms of number of genomes analysed in an academic publication so far, and reported results could be useful in monitoring the development of SARS-CoV-2.

## 1. Introduction

COVID-19 is the second largest pandemic we have encountered in the last century, with approximately 170 million diagnosed cases and more than 3.5 million deaths to date (WHO, 2021).^1^

 There is no accepted systemic drug therapy that can be used to treat COVID-19 other than a limited number of candidate molecules yet (Chan et al., 2021; Kathiravan et al., 2021; Golamari et al., 2021). But some vaccines that have been started to be used in many countries have become the greatest hope of humanity to return to their normal life that they had before the pandemic (Polack et al., 2020; Hosseini et al., 2021, Zhang et al., 2021). Scientists’ devoted studies on the virus, especially in the fields of microbiology, genomics and proteomics, underlie the success of the treatment and immunity options that are ready for use in a short time (Wu et al., 2020; Zost et al., 2020; Gorbalenya et al., 2020).

Over 80,000 publications indexed with the keyword “SARS-CoV-2” in the PubMed database show that^2^, within the 18-month period starting from the first definition, studies on this virus have provided knowledge about other pathogens or organisms. Certainly, one of the most important roles in the formation of this knowledge is the analytical ability that information technologies provide to scientists. It has become possible to observe the evolution, distribution, and genomic variations of the virus in real time with the combination of the widespread use of automation in the workflow of DNA sequencing and the information technologies that enable the processing of big data (Agbehadji et al., 2020; Shaffaf and Ghafar-Zadeh, 2021). The fact that even a couple of nucleotide exchanges in the virus genome could affect the spread of the virus, the progression of the disease and the success of immunity and treatment approaches increase the importance of these studies (Grubaugh et al., 2020; Isabel et al., 2020; Planas et al., 2021). 

GISAID (global initiative on sharing avian influenza data) platform (Elbe and Buckland-Merrett, 2017; Shu and McCauley, 2017) is established in 2008 to support virological studies.^3^ The platform shared the first SARS-CoV-2 genome sequence in EpiCoV^4^ on January 10th, 2020, and, as of April 5th, 2021, the number of submissions of SARS-CoV-2 isolate genome sequences in this database has exceeded 1 million.

Even though there are limited number of platforms or tools for monitoring the mutations that occur in the genome of SARS-CoV-2 during the pandemic, like NextSrain^5^ (Hadfield et al., 2018), and Pangolin^6^ (Rambaut et al., 2020), it is still a challenge for researchers to analyze this data and extract knowledge from this vast amount of genome data.

In our previous study with 30,366 genome sequences, we have reported the variations of SARS-CoV-2, date, and location they occurred, relationship of variations with each other and their effect on the primary protein structure (Uğurel et al., 2020). After a year passed, the genomic data exceeded 1 million submissions, and this led to the need to update the study and write the genomic chronicle of the virus.

In this study, we have analysed the genome of over 1 million SARS-CoV-2 isolates and presented results showing the distribution, direction, and relationship of variations that could enlighten the evolution and development of the virus.

This study is one of the most comprehensive SARS-CoV-2 genomic analysis studies in terms of the number of genomes analysed in a single study so far. Chronological analysis results presented here in this study would be useful in monitoring the evolution of the SARS-CoV-2 via analysing genomic variations and/or mutations and the possibility of its future development.
WHO (2021). Coronavirus disease (COVID-2019) Health Emergency Situation Dashboard [online]. Website: covid19.who.int [accessed 02 June 2021].
PubMed (2021). [online]. Website: https://pubmed.ncbi.nlm.nih.gov/?term=sars-cov-2&size=200 [accessed 02 June 2021].
GISAID (2020). Enabling rapid and open access to epidemic and pandemic virus data. [online] Website: https://www.gisaid.org/about-us/mission/[ accessed 25 April 2021].
EpiCoV (2021). Pandemic Coronavirus Causing COVID-19 [online]. Website: https://www.epicov.org/ [accessed 28 April 2021].
NextStrain (2021). Nextstrain: Analysis and Visualization of Pathogen Sequence Data [online] Website: https://nextstrain.org/ [accessed 28 April 2021].
Pangolin (2021). Phylogenetic Assignment of Named Global Outbreak Lineages [online] Website: https://cov-lineages.org/[accessed 28 April 2021].

## 2. Materials and methods

1,018,031 genomic sequences of SARS-CoV-2 isolates submitted on GISAID/ EpiCoV database (GISAID, 2020) by 7th April 2021 have been downloaded into our local database in FASTA file format for alignment and analysis. ODOTool is a strategy based local alignment tool, which standardizes all downloaded data, aligns nucleotide and amino acid sequences by modified Needleman–Wunsch algorithm, modified BLOSUM 62 scoring matrix and adjusted gap penalties, determines the SNVs and store all aligned genomic data and SNVs in our local database with annotations such as isolate name, location, collection, and submission dates, prior to further analyses (Uğurel et al., 2020). The downloaded genome sequences were aligned by ODOTool, based on the reference Wuhan strain NC_045512.2 (Wu et al., 2020). These 1,018,031 genomic sequences were pre-analysed, and 21,547 genomic sequences were not classified as with high coverage according to < 1% single nucleotide variations (SNV) as in the high coverage criteria implemented by GISAID and so were omitted in the further analysis. As the genome sequences collected between December 1st, 2019 and March 31st, 2021 were evaluated within the scope of the study, 9897 sequences entered without time annotations in months were also ignored in the chronological analysis. SNV of each genomic position in each isolate was computed by ODOTool and stored in local database for further analyses. After calculating the SNV numbers, the isolates were grouped monthly and diagrams were created.

## 3. Results and discussion

Since the first identification of COVID-19, devoted works of the scientists has resulted in production of tens of thousands of academic publications, proposal of thousands of drug candidates, development of hundreds of diagnostic kits and application of clinical vaccine/drug studies, and finally availability of some of authorized treatment and immunization options to ultimately end the COVID-19 pandemic. 

Undoubtedly, genome sequencing analyses were also among this intense effort to determine the genomic makeup of SARS-CoV-2 to support all these studies and also monitor the virus’s variations to possibly understand its evolution to overcome the disease. 

**Figure 1:**
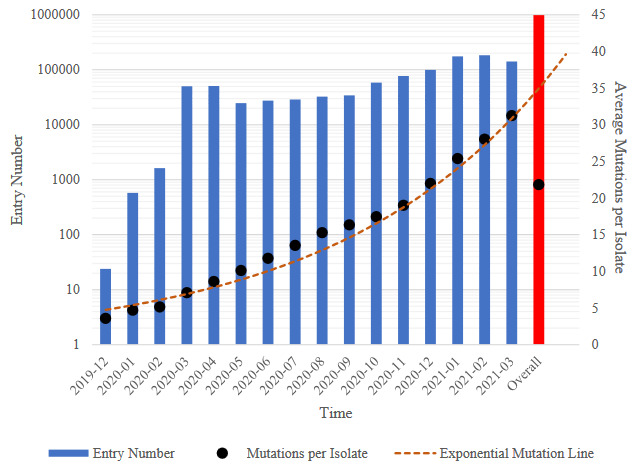
The temporal change of GISAID entry numbers of SARS-CoV-2 isolate genomes and average SNV numbers per isolates.

The number of sequenced SARS- CoV-2 genome were quite low at the beginning but gradually increased in parallel to the increase in the number of cases with the spread of pandemic all over the world. More than 1 million SARS-CoV-2 genomes were sequenced and submitted in open access databases in the 16-month period from December 2019 to March 2021. In this study, 1,018,031 SARS-CoV-2 genomic sequences were first analysed to remove low coverage genomes and sequences with the missing data entries required for the analyses performed. Considering the collection dates, there were only 24 isolate genomes available in December 2019, 57,060 isolates in March 2020 and the number reached to total of 1,018,031 isolates in March 2021 (Figure 1). Figure 1, which shows that the evolutionary change is as expected, also reveals the nucleotide exchange rate of the virus numerically and temporally.

**Table:** Summary of SNVs seen over 10% frequency of isolate genomes that are studied in this article.

Genome position	Nucleotide exchange	Frequency %	Region	Amino acid exchange
241	C→T	93.51	5’UTR	-
445	T→C	14.06	ORF1a/Nsp1	silent
913	C→T	28.51	ORF1a/Nsp2	silent
1059	C→T	17.96	ORF1a/Nsp2	T85I
3037	C→T	96.03	ORF1a/Nsp3	silent
3267	C→A	28.91	ORF1a/Nsp3	T183I
5388	C→T	28.56	ORF1a/Nsp3	A890V
5986	C→T	28.82	ORF1a/Nsp3	silent
6286	C→T	14.36	ORF1a/Nsp3	silent
6954	T→C	28.23	ORF1a/Nsp3	I1412T
14408	C→T	94.12	ORF1b/Nsp12	P314L
14676	C→T	28.79	ORF1b/Nsp12	silent
15279	C→T	28.71	ORF1b/Nsp12	silent
16176	T→C	28.62	ORF1b/Nsp12	silent
21255	G→C	14.00	ORF1b/Nsp16	silent
22227	C→T	14.45	S	D215G
23063	A→T	29.76	S	N501Y
23271	C→A	28.69	S	A570D
23403	A→G	95.13	S	D614G
23604	C→A	30.49	S	P681H
23709	C→T	28.86	S	T716I
24506	T→G	28.66	S	S982A
24914	G→C	28.75	S	D1198H
25563	G→T	23.28	ORF3a	Q57H
26801	C→G	13.92	M	silent
27972	C→T	28.75	ORF8	silent
28048	G→T	28.41	ORF8	R52I
28111	A→G	28.35	ORF8	Y73C
28280	G→C	27.19	N	D3L
28281	A→T	27.35	N	D3L
28282	T→A	27.43	N	D3L
28881	G→A	46.65	N	R203K
28882	G→A	46.45	N	R203K
28883	G→C	45.09	N	G204R
28932	C→T	14.42	N	A220V
28977	C→T	29.15	N	S235F
29645	G→T	13.71	ORF10	V30L

When all SARS-CoV-2 isolate genomes in our database were aligned and compared with the reference SARS-CoV-2 genome sequence (NC_045512.2), total of 215,393,375 SNV were detected. When SARS-CoV-2 genome sequences are examined, it was observed that the SNV average per isolate increases exponentially and directly proportionally over the time. In the studies conducted in June and August 2020, the SNV average was reported to be 7.23 (Mercatelli and Giorgi, 2020) and 8.01 (Eskier et al., 2021), respectively. It was calculated as 21.83 SNV per isolate when all isolate genomes were examined in this study (Fig 1). SNV average is observed to reach 31.25 just in March 2021.

**Figure 2:**
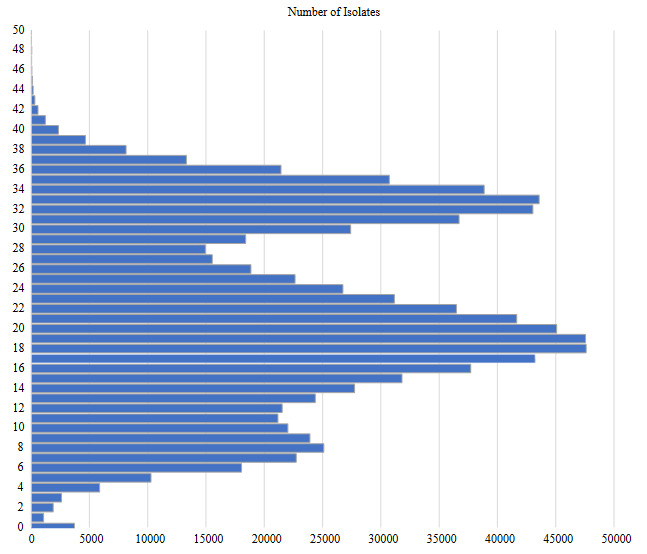
Histogram of number of isolates vs. number of SNVs.

The isolate genome sequences were grouped by the number of SNVs they harbour. As a result of this grouping, isolates harboured between 10 and 35 SNVs comprise more than 80% of all isolates, while the proportion of isolates harboured >50 SNVs is less than 0.3% of all isolates. The histogram of the number of isolates grouped according to the number of SNVs they carry is given in Figure 2. 

**Figure 3:**
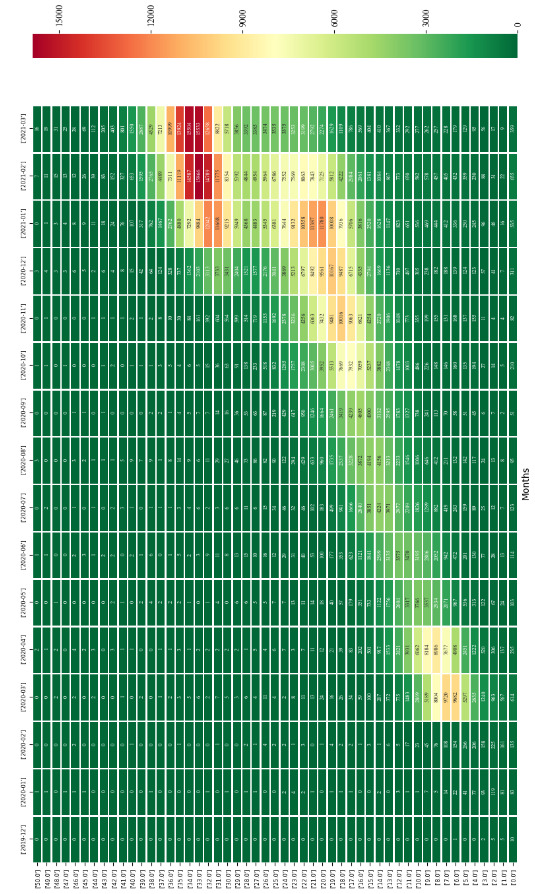
The temporal distribution of isolate numbers by harboured SNVs. Rows represent harboured SNV numbers and columns represent the months.

Considering the number of SNV harboured by the isolates, it is a remarkable finding that a total of 3715 isolate genome sequences out of sequences analysed in this study were the same as the sequence from Wuhan strain (NC_045512.2) (Figure 2). The vast majority of 3715 genome sequences without any SNV were submitted from Argentina (838), Italy (464), Hong Kong (291), France (274) and United Kingdom (233). When the genomes with no SNVs from these regions were rated over the total number of isolate genomes, Argentina 37%, Italy 3%, Hong Kong 24.8%, France 1.8%, and United Kingdom 0.6%. Despite more than 1 year, no nucleotide changes were observed in 1549 of the 501.287 sequences submitted between January and March 2021 (Fig 3). 

The data of isolate genome sequences and the number of SNVs were also evaluated chronologically and a heat mapped was created by months. It is clearly seen that the hot areas in which these isolates are heat mapped by months overlapped with three periods: March–April 2020, November 2020–January 2021, and January–March 2021 (Figure 3). Data in Figure 3 is in consistency with these periods where the global spread of the epidemic increased.^7^
WHO (2021). WHO Coronavirus (COVID-19) Dashboard [online]. Website: https://covid19.who.int/ [accessed 30 May 2021].


Despite the identification of so many SNVs in the past 16 months, changes in the virus’s proteins are very limited. The reason for this situation can be the codon usage bias (CUB), which can have specific consequences in different organisms, as there are several irregularly used synonymous codons that encode most of the amino acids (Belalov and Lukashev, 2013). 

CUB is explained with 2 basic conditions: translational selection, meaning the selection of the most suitable codon for translation, and the mutational pressure gained by different probabilities of different types of substitutions, such as GC content, methylation of deoxycytidine (C-phosphate-G), or subsequent deamination (C-T substitution) (Bulmer, 1987; Sharp et al., 1993; Belalov and Lukashev, 2013). Cytosine deamination is a known process that is an important source of synonymous mutations (Duncan and Miller, 1980) managing the GC contents of RNA viruses (Pyrc et al., 2004). Cytosine deamination has been observed in all coronavirus genomes and suggested as a significant biochemical impact on coronavirus evolution (Woo et al., 2007).

**Figure 4:**
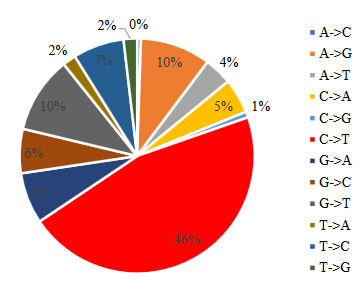
The distribution of nucleotide changes in SARS-CoV-2 isolate genomes.

When it comes to SARS-CoV-2, our studies show that, even the compositional value of C is the lowest by 18.3% (Hou, 2020) in nucleotide composition of SARS-CoV-2 Wuhan-Hu-1, the C is also the most mutated nucleotide. A total of 46% of all SNVs stand out prominently as variations in the C→T direction (Figure 4). 

Besides random SNVs, some SNVs can spread from an emerging lineage, reaching a predominant or substantial proportion among isolates worldwide. But inconsistency in the distribution of the numbers of the isolate genome sequences submitted from different regions raise an important bias issue to consider. For example, the number of genomes submitted only from United Kingdom is higher than all continents, including Continental Europe and North America. This makes it difficult to detect SNVs that occur locally but may be important in the spread or aspect of the epidemic. Therefore, frequent SNVs should be additionally evaluated regionally or locally. 

**Figure 5:**
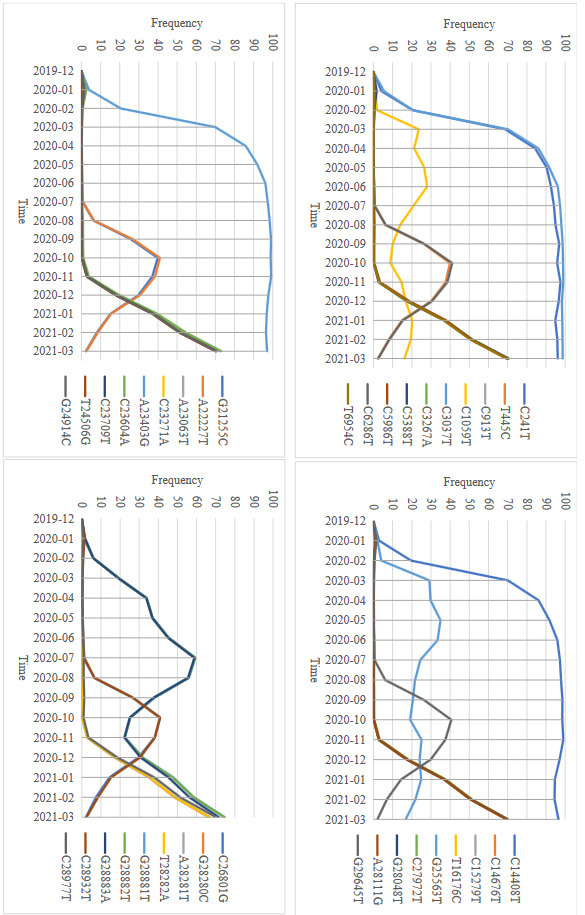
Monthly changes of 38 SNVs, which are seen in more than 10% frequency among all isolates.

**Figure 6:**
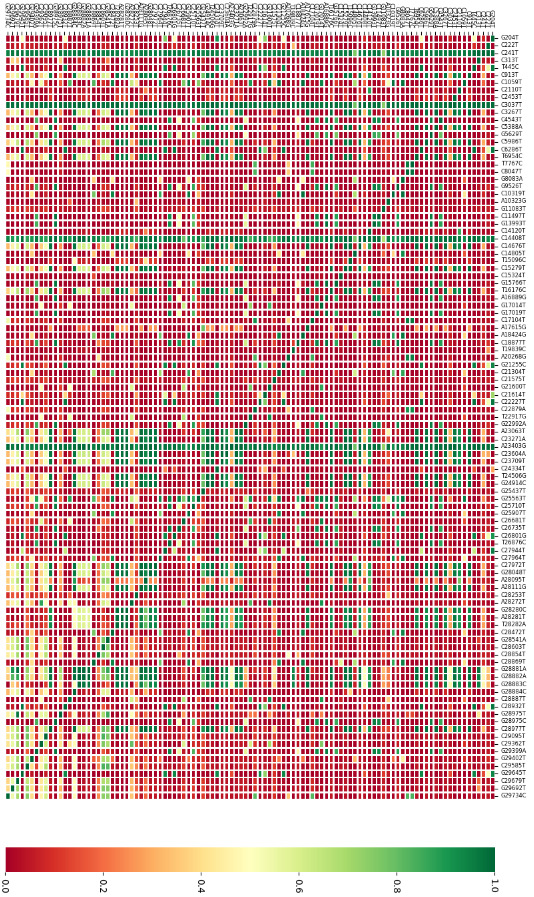
Heatmap of the co-existence of 102 SNVs, which are seen in more than 2% frequency among all isolates, against each other. Each row shows the co-existence of the SNV in that row and the other SNVs harboured in the isolates carrying it.

In the light of all this information, repetitively and consistently seen SNVs were discussed in this study to show their rate of spread and coexistence (Figures 5 and 6). Although random SNVs are seen throughout the SARS-CoV-2 genome, SNVs at 1314 positions are seen in more than 1% of all isolates, SNVs at 102 positions are seen in more than 2% of all isolates, and SNVs at 38 positions are seen in more than 10% of all isolates. Genomic positions, nucleotide exchanges, genome regions, and amino acid exchanges of 38 SNVs that seen over 10% frequency in all isolate genomes are presented in Table. It should also be noted that the presence of reported SNVs here in this study has also been discussed in many aspects in some other previous studies (Uğurel et al., 2020; Pachetti et al., 2020; Wang et al., 2020; Phan et al., 2020; Mercatelli and Giorgi, 2020; Eskier et al., 2021; Alkhansa et al., 2021; Ozono et al., 2021; Wang et al., 2021, Qin et al., 2021).^8^
NextStrain (2020). Nextstrain: analysis and visualization of pathogen sequence data [online] Website: https://nextstrain.org/[ accessed 17 May 2020].


The rapid increase in sequenced isolate genomes has brought out the need for a phylogenetic classification system, depending on the effects of the mutations they carry and their co-existence. Although there are different phylogenetic classifications, the basic principle is based on the coexistence of SNVs in isolates (Zehender et al., 2020; Rambaut et al., 2020). In this study, beyond the variants, SNVs are presented individually in terms of frequency changes by time. While some SNVs spread quite rapidly, some SNVs appear to spread very slowly, even decreasing over the time (Figure 5). 

Frequencies of C241T, C3037T, C14408T, and A23403G reached to 20% in February 2020, 85% in April 2020, and 96% in June 2020 (Figure 5). A23403G on S protein region (D614G) was reported to be responsible from rapid spread (Grubaugh et al., 2020). 

G28881A, G28882A and G28883C SNVs correspond to R203K, G204R substitutions on N protein, C1059T correspond to T85I on ORF1ab/Nsp2, and C25563T, which corresponds to Q57H substitutions on ORF3a protein, present a fluctuating graph (Figure 5). 

Another group comprises T445C, C6286T, G21255C, A22227T, C26801G, C28932T, G29645T was noted for the first time in August 2020 with a remarkable ratio of 6%. After peaking at 40% in October 2020, they gradually decreased and finally fell to 2% in March 2021. 

According to Figure 5, the remaining 21 SNVs with the equivalent uptrend can be regarded as a single group. 6 SNVs that cause substitutions on S protein, especially A23063T (N501Y), are reported as key mutations and associated with these 21 SNVs (Volz et al., 2021; O’Toole, 2021). In this study, we observed that frequency of these 21 SNVs increased with a very high acceleration just like the C241T, C3037T, C14408T, and A23403G group and reached 70% frequency in March 2021. 

Some of SNVs associated with the current spread of the epidemic such as A23403G, A23063T (Volz et al., 2021; O’Toole, 202; Grubaugh et al., 2020) are listed in Table. However, the mutation that will be responsible for the spread and effects of the virus in the upcoming period may be among one or more of the SNVs currently seen below 10% frequency. 

In Figure 6, association between 102 SNVs, which are harboured at least 2% of all isolates including the 38 SNVs, was analysed in detail in the present study. The relationship between SNVs should be considered in two directions as shown in an example: isolates harbouring any of the 102 SNVs also harbour the C241T variation, but isolates carrying C241T have over 20% association with only 27 out of 102 SNVs. The association between 102 SNVs has been ranked from 0 to 1 and heat-mapped to be easily traceable (0: red, 1: green) (Figure 6). The association data in Figure 6 emerged as a result of analyses performed in this study may present significant observations in terms of monitoring mutations.

## Conclusion

The current study is remarkable in terms of the number of SARS-CoV-2 genome analysed in an academic publication. In the past 18 months, the pandemic continues unabated. During this time, the genomic sequence data from SARS-CoV-2 isolates exceeded 1 M submissions, and it is obvious that this number will be higher by the time of writing this paper. Analyses performed in this study present a genomic chronicle for the SARS-CoV-2 genome sequence. 
